# Retraction: IFITM3 promotes hepatocellular carcinoma invasion and metastasis by regulating MMP9 through p38/MAPK signaling

**DOI:** 10.1002/2211-5463.13751

**Published:** 2023-12-27

## Abstract

IFITM3 promotes hepatocellular carcinoma invasion and metastasis by regulating MMP9 through p38/MAPK signaling. Jiaqi Min, Qian Feng, Wenjun Liao, Yiming Liang, Chengwu Gong, Enliang Li, Wenfeng He, Rongfa Yuan, Linquan Wu, *FEBS* *Open Bio*, 8: 1299–1311. https://doi.org/10.1002/2211-5463.12479

The above article, published online on 14 June 2018 in Wiley Online Library (wileyonlinelibrary.com), has been retracted by agreement between the journal Editor‐in‐Chief Miguel De la Rosa, the corresponding author, FEBS Press, and John Wiley and Sons Ltd. The retraction has been agreed due to the identification of duplication and repositioning of images in Figs 2C–E and 3C,D and excessive editing (high contrast) of Western blot images throughout the article. The authors were unable to provide the original raw Western blot data upon request. Given the extent of the identified issues, the editors have lost confidence in the data presented and have therefore decided to retract it.

IFITM3 promotes hepatocellular carcinoma invasion and metastasis by regulating MMP9 through p38/MAPK signaling. Jiaqi Min, Qian Feng, Wenjun Liao, Yiming Liang, Chengwu Gong, Enliang Li, Wenfeng He, Rongfa Yuan, Linquan Wu, *FEBS* *Open Bio*, 8: 1299–1311. https://doi.org/10.1002/2211-5463.12479

The above article, published online on 14 June 2018 in Wiley Online Library (wileyonlinelibrary.com), has been retracted by agreement between the journal Editor‐in‐Chief Miguel De la Rosa, the corresponding author, FEBS Press, and John Wiley and Sons Ltd. The retraction has been agreed due to the identification of duplication and repositioning of images in Figs 2C–E and 3C,D and excessive editing (high contrast) of Western blot images throughout the article. The authors were unable to provide the original raw Western blot data upon request. Given the extent of the identified issues, the editors have lost confidence in the data presented and have therefore decided to retract it.

